# Population pharmacokinetics and dose optimization of vancomycin in neonates

**DOI:** 10.1038/s41598-021-85529-3

**Published:** 2021-03-17

**Authors:** Soon Min Lee, Seungwon Yang, Soyoung Kang, Min Jung Chang

**Affiliations:** 1 Department of Pediatrics, Yonsei University College of Medicine, Seoul, Korea; 2 Department of Pharmacy, Yonsei Institute of Pharmaceutical Sciences, Yonsei University, Incheon, Republic of Korea; 3 Department of Pharmaceutical Medicine and Regulatory Science, Yonsei University, Veritas Hall D #214, Yonsei University International Campus, Songdogwahak-ro 85, Yeonsu-gu, Incheon, Korea

**Keywords:** Computational biology and bioinformatics, Diseases, Medical research

## Abstract

The pharmacokinetics of vancomycin vary among neonates, and we aimed to conduct population pharmacokinetic analysis to determine the optimal dosage of vancomycin in Korean neonates. From a retrospective chart review, neonates treated with vancomycin from 2008 to 2017 in a neonatal intensive care unit (NICU) were included. Vancomycin concentrations were collected based on therapeutic drug monitoring, and other patient characteristics were gathered through electronic medical records. We applied nonlinear mixed-effect modeling to build the population pharmacokinetic model. One- and two-compartment models with first-order elimination were evaluated as potential structural pharmacokinetic models. Allometric and isometric scaling was applied to standardize pharmacokinetic parameters for clearance and volume of distribution, respectively, using fixed powers (0.75 and 1, respectively, for clearance and volume). The predictive performance of the final model was developed, and dosing strategies were explored using Monte Carlo simulations with AUC_0–24_ targets 400–600. The patient cohort included 207 neonates, and 900 vancomycin concentrations were analyzed. Only 37.4% of the analyzed concentrations were within trough concentrations 5–15 µg/mL. A one-compartment model with first-order elimination best described the vancomycin pharmacokinetics in neonates. Postmenstrual age (PMA) and creatinine clearance (CLcr) affected the clearance of vancomycin, and model evaluation confirmed the robustness of the final model. Population pharmacokinetic modeling and dose optimization of vancomycin in Korean neonates showed that vancomycin clearance was related to PMA and CLcr, as well as body weight. A higher dosage regimen than the typical recommendation is suggested.

## Introduction

Neonates, especially premature infants, are susceptible to gram-positive infections^1^. According to the Korean Neonatal Network, the overall incidence of sepsis among low-birth weight infants was 21%, with gram-positive organisms being the most prevalent organisms: coagulase-negative *Staphylococcus* is most common, followed by *Staphylococcus aureus*^2^. Methicillin-resistant *S. aureus* (MRSA) accounts for 80% of all isolated *S. aureus* cases in intensive care units and has become a threat to critically ill populations in Korea^3^. Neonates with suspected sepsis typically receive vancomycin.

Neonates exhibit major and rapid physiological changes in drug distribution, metabolism, excretion, and absorption (for drugs after parenteral administration)^4^. More extensive inter- and intra-individual variability in pharmacokinetics has been described for neonates, compared to adults, and a larger variability in drug disposition and a poor relation between the dose administered and the concentration achieved in neonates has been recorded for most drugs^5,6^.

Vancomycin, a glycopeptide antibiotic, has the potential to cause severe nephrotoxicity and ototoxicity. Historically, vancomycin dosing has been titrated to obtain serum trough concentrations of 10 to 15 mg/L for infections and 15 to 20 mg/L for severe infections, such as bacterial meningitis^7,8^. Several proposed vancomycin dosing regimens are currently available for neonates; however, target trough concentrations are difficult to attain in clinical practice^9^. Despite the widespread use of vancomycin and therapeutic drug monitoring in neonates, controversy exists regarding optimal pharmacokinetics (PK)/pharmacodynamics (PD) targets, dosing, and monitoring^1^. There is also evidence of correlations between serum vancomycin concentrations and clinical cure, microbiological cure, and nephrotoxicity in neonates^10,11^.

Although several population pharmacokinetic models of vancomycin exist, only a few individualized, model-based dosing algorithms in neonates are available^9,12,13^. Population PK modeling and simulation approaches allow us to characterize population averages, to assess the inter- and intra-subject variability of pharmacokinetic parameters, and to identify the optimal dosing of antibiotics. Accordingly, this study aimed to conduct a population pharmacokinetic analysis to obtain pharmacokinetic data for vancomycin in Korean neonate infants and to develop an optimal vancomycin dosing strategy using model simulation.

## Results

### Patients

The demographic and clinical characteristics of the 207 neonates are described in Table 1. The median gestational age was 31.5 weeks; the median birth weight was 1.5 kg.

**Table 1:** Demographic and clinical characteristics of the study population.

Characteristic	N = 207
Male, n (%)	103 (50)
**Gestational age, weeks**	31.5 (23.3–41.5)
< 28 weeks, n (%)	68 (33)
28–31 weeks, n (%)	38 (18)
32–36 weeks, n (%)	34 (16)
≥ 37 weeks, n (%)	67 (32)
**Birth weight, kg, mean ± SD**	1.5 (0.5–5.4)
< 1.5 kg, n (%)	75 (36)
< 2.5 kg, n (%)	51 (25)
≥ 2.5 kg, n (%)	81 (39)
Apgar score at 1 min	4 (0–9)
Apgar score at 5 min	7 (0–10)
Post-natal age at examination, weeks	2.3 (0–16.4)
Postmenstrual age at examination, weeks	35.6 (24.0–48.4)
Body weight at examination, kg	1.8 (0.5–5.9)
Body length at examination, cm	41 (27–54)
Blood urea nitrogen, mg/dL	8.7 (1.3–76.4)
Serum creatinine, mg/dL	0.5 (0.2–2.6)
Creatinine clearance rate, mL/min/1.73 m^2^	50.3 (6.8–140.3)
Serum protein	4.9 (2.9–6.9)
Serum albumin level, g/L	3.2 (1.8–4.7)
AST, IU/L	25 (13–1838)
ALT, IU/L	10 (3–547)
C-reactive protein, mg/L	1.5 (0.1–123.3)
Vancomycin concentration, mcg/mL	18.5 (1.3–160.0)

Data are presented as median (range).

*AST* aspartate transaminase, *ALT* alanine aminotransferase.

In total, 900 vancomycin concentrations were analyzed. Vancomycin treatment was administered at a median post-natal age (PNA) of 2.3 weeks and postmenstrual age (PMA) of 35.6 weeks. The dosage regimens administered to patients varied, with most patients receiving a dose of 10 mg/kg for 1 h every 8 or 12 h. Only 37.4% (337/900) were within the therapeutic range.

### Pharmacokinetic modeling

The observed vancomycin concentration–time profiles were best described by a one-compartment model with first-order elimination. The model-derived pharmacokinetic parameters were CL and V. Interindividual variability was best explained by an exponential model that estimated CL and V; the model showed a correlation between total clearance (CL) and volume of distribution (V) [△ objective function value (OFV) = – 47.9]. Residual variability was best described by a combined additive and proportional model.

The final population PK models of vancomycin in neonates were best explained by the following equations:

1 $$ {\text{CL}} = 2.09 \times \left( {\frac{{{\text{WT}}}}{70}} \right)^{{{0}{\text{.75}}}} \times { }\left( {\frac{{{\text{PMA}}}}{31.7}} \right)^{0.795} \times { }\left( {\frac{{{\text{CLcr}}}}{50.3}} \right)^{0.741} $$

2 $$ {\text{V}} = 45.6 \times \left( {\frac{{{\text{WT}}}}{70}} \right)^{1} $$

With the allometric size approach mentioned in the methods, PMA and CLcr on CL improved the population PK model of vancomycin in neonates (△OFV = –809.8). The final population pharmacokinetic model parameters for vancomycin and the bootstrap results are provided in Table 2.

**Table 2:** Final population pharmacokinetics model parameters for vancomycin in neonates.

Parameters	Estimate (%RSE) [%shrinkage]	Bootstrap median (95% CI)^a^
Final model	TVCL = $\uptheta {1}\times {\left(\frac{\mathrm{WT}}{70}\right)}^{0.75}\times {\left(\frac{\mathrm{PMA}}{31.7}\right)}^{\uptheta {2}}\times {\left(\frac{\mathrm{CLcr}}{50.3}\right)}^{\uptheta {3}}$ TVV = $\uptheta {4}\times {\left(\frac{\mathrm{WT}}{70}\right)}^{1}$	
θ_1_	2.09 (3%)	2.07 (1.94, 2.21)
θ_2_	0.795 (26%)	0.817 (0.396, 1.241)
θ_3_	0.741 (6%)	0.739 (0.641, 0.820)
θ_4_	45.6 (5%)	45.7 (41.6, 50.6)
**Interindividual variability (ω)**		
$\upomega$_CL_	0.123 (26%) [21%]	0.130 (0.068, 0.217)
$\upomega$_V_	0.260 (25%) [35%]	0.296 (0.190, 0.425)
**Residual variability (σ)**		
σ_proportional_ (%CV)	58.3% (8%)	58.6 (53.7, 63.8)
σ_additive_ (mg/L)	2.015 (18%)	2.001(1.560, 2.296)

^a^95% CI estimated from 1000 resampled data sets using the final population (993 successful run).

*TVCL* typical value of clearance (L/h), *TVV* typical value of volume of distribution (L), *CLcr* creatinine clearance (calculated by the Schwartz equation), *PMA* postmenstrual age, *WT* body weight, *ω*_*CL*_ interindividual variability of clearance, *ω*_*v*_ interindividual variability of volume of distribution, *σ*_*proportional*_ proportional residual error, *σ*_*additive*_ additive residual error.

**Figure 1:**
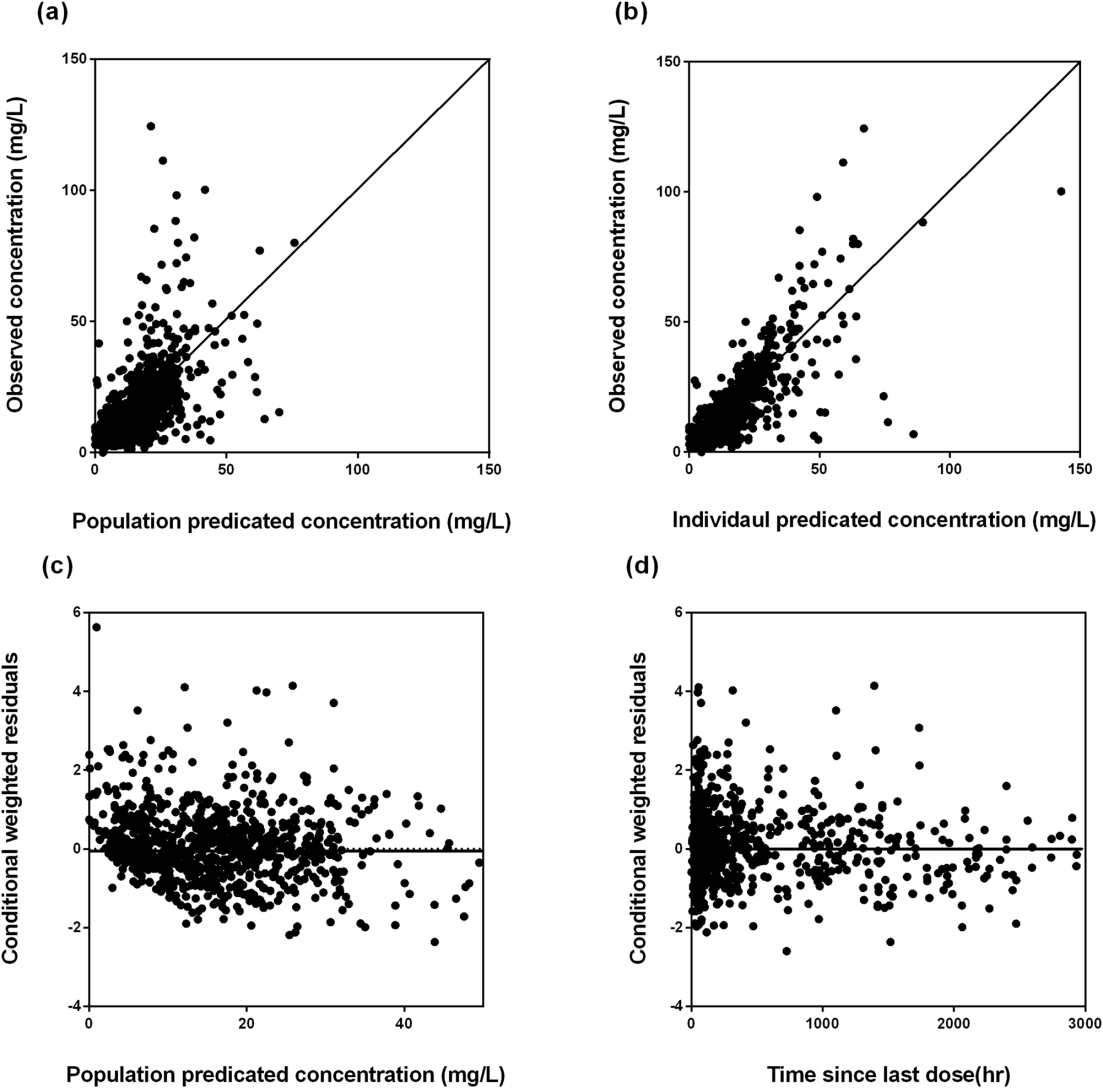
Goodness-of-fit plots of the final population pharmacokinetics (PK) model for vancomycin. Observed vancomycin concentrations vs*.* **(a)** population-predicted concentrations and **(b)** individual-predicted concentrations. Conditional weighted residuals vs. **(c)** population-predicted concentrations, and **(d)** time since last dose.

**Figure 2:**
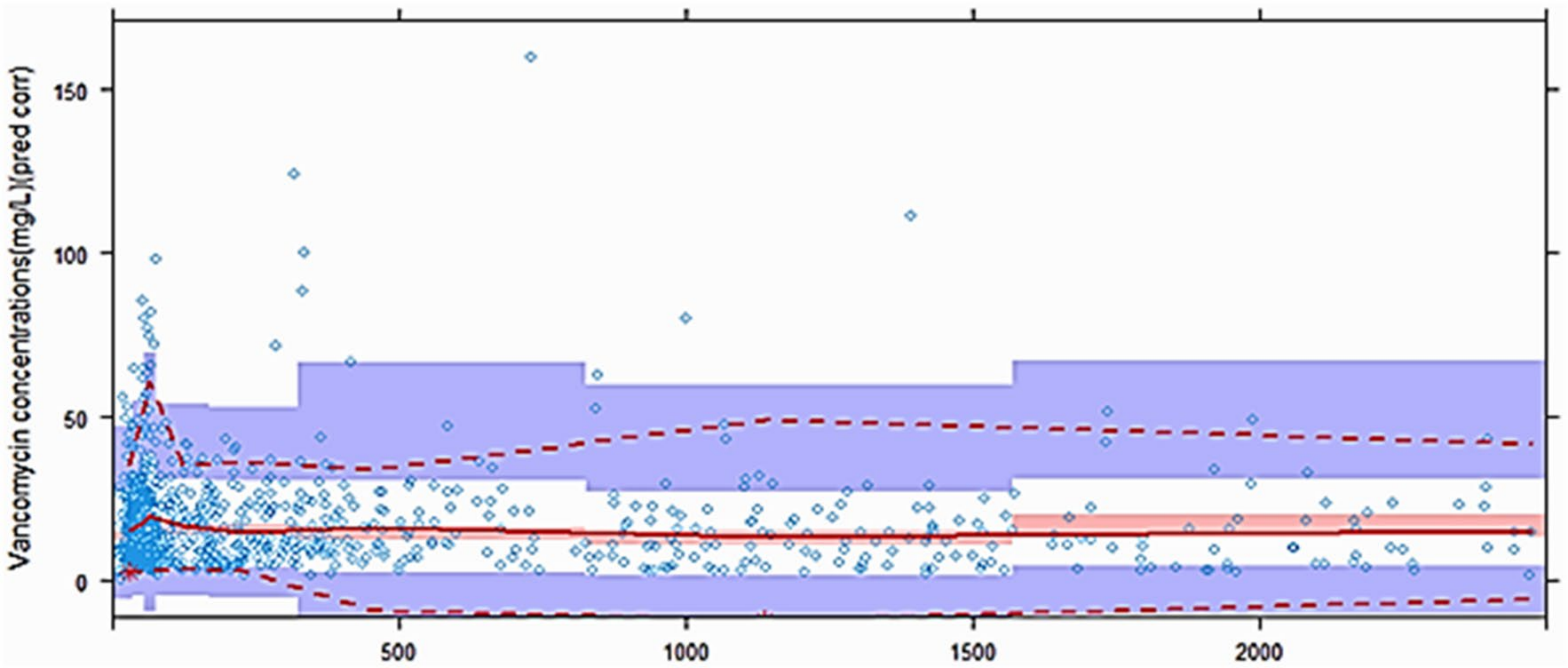
Prediction-corrected visual predictive check for vancomycin. A thousand simulations were performed. Prediction-corrected observed concentrations are shown as open circles. The middle solid, lower dashed, and upper dashed lines represent the median, 2.5th, and 97.5th percentiles for the observed data, respectively. The shaded areas represent a 95% confidence interval for a simulated predicted median, 2.5th, and 97.5th percentiles constructed from 1000 simulated datasets of individuals from the original dataset.

Model diagnostics showed adequate goodness-of-fit for the final vancomycin PK model. There was good agreement between the observed and population-predicted/individual-predicted values for vancomycin serum concentrations (Fig. 1a,b). Figure 1c,d show that the conditional weighted residual vs. population-predicted values and time since last dose were equally distributed around zero and did not show any trends. The nonparametric bootstrap analysis results are shown in Table 2. The prediction corrected (pc)-visual predictive check (VPC) results revealed the good predictive performance of the final population PK model for vancomycin (Fig. 2). After 1,000 simulations, the observed median, 2.5th, and 97.5th data were adequately captured by the corresponding simulation-based prediction intervals and 95% confidence intervals.

### Simulation and dose optimization

The simulation of peak and trough concentrations at 96 h after the administration of vancomycin at 10, 15, and 20 mg/kg/dose every 6, 8, 12, and 24 h was performed based on target AUC_0–24_ values ≥ 400. The optimized dose recommendations based on the simulated results are listed in Table 3. For example, if patients are PMA 32 weeks, with a CLcr of 40 mL/min/1.73 m^2^, a steady-state trough concentration of 15 mg/kg/dose q12h is recommended.

**Table 3:** Dose recommendations for vancomycin in neonates.

Dose (mg/kg)	PMA (weeks)						
CLcr (mL/min/1.73 m^2^)	< 24	24–27	28–31	32–35	36–39	40–43	44 ≤
< 15	10 q24h			10–15 q24h		15 q24h	
15–30	10 q24h		15 q24h	15 q24h	10 q12h	10–15 q12h	10 q8h
30–60	10 q12h		10 q8–12 h		15 q8–12 h	15 q8h	
60–90	10 q8–12 h	10 q8h	15 q8–12 h	15 q8h	15–20 q8h	20 q8–12 h	20 q8h
90 ≤	15 q12h	15–20 q12h	20 q12h	20 q8–12 h	20 q8h		25 q8h

*CLcr* creatinine clearance, *PMA* postmenstrual age.

## Discussion

Here, we aimed to determine the population PK of vancomycin in neonates and to optimize doses to better reflect clinical situations based on trough concentrations. This is the first study to evaluate dose optimization based on population PK modeling and to consider the clinical situations of limited samples for TDM and nephrotoxicity. The observed plasma vancomycin concentrations were well described; the recommended doses were predicted by simulation using the final population PK model. The study results were based on a one-compartment model, not a two-compartment model as in other studies ^14,15^. However, in support of our results, many studies have also shown that vancomycin PK in neonates is best described by a one-compartment model^13,16^.

Here, a vancomycin 10 mg/kg/dose was administered according to PMA and PNA recommended by Neofax^7^. Most patients were administered 10 mg/kg/dose q8h or q12h; lower or higher doses were administered based on the individual patients. With an increase in PMA or CLcr, vancomycin CL also increased; thus, allometric and isometric scaling using weights for both CL and V was applied to reflect neonatal characteristics. Our results were similar to those in previous studies showing that PMA and renal function are related to vancomycin PK parameters in neonates^12,13,16,17^. Tseng et al. studied vancomycin PK in 76 neonates. They reported that PMA and SCr were related to vancomycin CL and performed allometric scaling using weights, although centering was additionally performed by dividing the weights by the median weight of the neonates^16^. Li et al. also tried to build a covariate model in vancomycin population PK modeling, and allometric scaling by weights for CL and V was also performed. Therein, SCr was inversely related to vancomycin CL^13^. As vancomycin is primarily excreted by the kidneys, kidney maturation is crucial for vancomycin dose determination. Accordingly, Rhodin et al. studied factors correlated with human renal function maturation and found that PMA is more strongly related to renal function maturation than PNA^17^, which other have also reported ^18–21^. In our study, the power model best explained the relationship between CL and PMA. The Hill equation was proposed by previous studies to describe the relationship between CL and PMA^22,23^. We also tried a sigmoidal model when describing maturation; however, because of the high value of variability and the limited range of PMA, we could not select that model as a final model. Also, the maturation of renal function was reported to reach about 62 weeks PMA ^24^ but the range of PMAs of our study were 24.0–48.4 weeks. Therefore, this is one of the causes why the result of our study could not explain the sigmoidal model. Anderson et al. compared linear, exponential, and sigmoidal models on PMA, and they reported that a sigmoidal model best described the correlation between PMA and CL. However, before 50 weeks, they showed near linear correlations^25^. Other studies also used power model to describe the age-effect of vancomycin population PKs in neonates instead of sigmoidal model^16,26,27^. Therefore, based on the definition of neonates within 4 weeks after birth, a sigmoidal model might not show the best fit.

In contrast to previous findings, we found that CLcr is more relevant to vancomycin CL than SCr^12,13,16^. SCr cannot reflect renal function directly because CLcr is also affected by age and weight. However, vancomycin PK parameters in neonates were usually adjusted by allometric scaling of weight: this might be why previous studies reported SCr to be related to vancomycin CL rather than CLcr. Here, we calculated CLcr using the Schwartz equation^28^, which better reflects the clinical situation of newborns.

There are numerous approaches to determining the adequate dosage regimen of vancomycin in neonates. To reflect PD and PK, area-under-the-curve concentrations versus time to the minimum inhibitory concentrations (AUC: MIC) can be used. Currently, AUC_24_/MIC ≥ 400 for MRSA infections provides sufficient treatment^16,29,30^ Tseng et al. reported that there is a positive relationship between AUC_24_ and trough concentrations at a steady state (C_ss,trough_) (r^2^ = 0.74) and that target trough concentrations 10‒14.9 μg/mL could achieve AUC_24_/MIC > 400 to ensure the efficacy and minimize nephrotoxicity and ototoxicity in neonates^16^. Frymoyer et al. reported that trough concentrations around 10 mg/L can achieve AUC_0-24_/MIC ≥ 400^31^. Another study suggested that C_ss,trough_ 5–15 μg/mL could satisfy AUC_0-24_/MIC ≥ 400 with less toxicity^12^. However, AUC-guided dosing uses the MIC of *S. aureus*, and vancomycin MIC in neonates can be more variable. A MIC of 1 mg/L is typically used for the AUC-guided dosing of vancomycin, and steady-state trough concentrations are used to predict the efficacy and safety of vancomycin^32–34^. As there is an increase in dose recommendation in neonates based on AUC-guided dosing, we also simulated target AUC_0–24_ values ≥ 400 when MIC ≤ 1. The recommended dose optimization was slightly higher than Neofax. We suggest a therapeutic range for vancomycin C_ss,trough_ of 5–15 μg/mL, as this range was highly predictive of AUC_0-24_/MIC ≥ 400^12^. Also, another study reported that C_ss,trough_ 15–20 μg/mL had higher nephrotoxicity in neonates^16^. A lower target than that of adults could be adequate for neonates, different from Neofax^7^. For example, neonates with PMA of 36 weeks and PNA of 10 days with bacteremia by MRSA and with CLcr 50 mL/min/1.73 m^2^, our model suggests 15 mg/kg q12h, whereas Neofax suggests 10 mg/kg q12h.

Although the final model in this study showed robustness, there were some limitations. This is a retrospective observational study, and only peak and trough concentrations were sampled for TDM. However, it is challenging to perform multiple tests on neonates, and 900 samples is considered substantial.

## Conclusion

This is the first study, to our knowledge, on vancomycin population PK modeling and dose optimization in a large number of Korean neonates. PMA and CLcr were related to vancomycin CL. Based on a C_ss,trough_ of 5–15 μg/mL, a higher dosage regimen that that currently employed is recommended. A future long-term study is necessary to confirm the efficacy and safety of the newly recommended dosage regimen.

## Materials and methods

### Study design and patient eligibility

The data of neonates admitted to the Gangnam Severance Hospital neonatal intensive care unit (NICU) from January 2008 to April 2017 who were treated with vancomycin for more than 24 h and had at least one steady-state plasma concentration measurement were retrospectively collected. Neonates were excluded if they developed acute kidney injury, including urine output < 1 mL/kg/h or serum creatinine ≥ 1.5 mg/dL, before the initiation of vancomycin administration. In total, 900 vancomycin concentrations from 207 neonates were included. The study protocol was approved by the institutional review board of Gangnam Severance Hospital (No. 3–2019-0206), Seoul, Korea, and the manuscript followed STROBE statement. The need for informed consent was waived by the institutional review board because the data were retrospectively collected. All methods were performed in accordance with the relevant guidelines and regulations.

### Data collection

All data were collected from electronic medical records. Patient characteristics included date of birth, sex, weight at the time of birth, weight at the time closest to vancomycin initiation, height at the time of birth, gestational age, post-natal age (PNA), postmenstrual age (PMA), type of delivery, APGAR 1 min of age, APGAR 5 min of age, blood urea nitrogen, serum creatinine (SCr), creatinine clearance (CLcr) calculated using the Schwartz equation [CLcr (mL/min/1.73 m^2^) = [length (cm) $\times$ k]/Scr (k = 0.45 for infants 1 to 52 weeks old)]^28^, protein, albumin, aspartate transaminase (AST), alanine aminotransferase (ALT), and c-reactive protein (CRP). Vancomycin dosing and plasma level information were as follows: the date of starting vancomycin treatment, vancomycin dosage, peak plasma concentrations of vancomycin, and trough plasma concentrations of vancomycin. The vancomycin dosages administered to neonates followed the Neofax recommendations^7^. According to Neofax, the dosing interval of 10 mg/kg/dose vancomycin should be determined by PMA and PNA. The peak vancomycin concentration was sampled 1 h after stopping vancomycin administration; trough concentrations were measured 0.5 h before the next dose. When there was more than one laboratory result or none on the same day as the plasma concentration sampling, the closest results were used in the analysis.

Serum vancomycin concentrations were analyzed via the chemiluminescent microparticle immunoassay (CMIA) (Abbott ARCHITECTi, Abbott Laboratories, Abbott Park, IL, USA). This method has a coefficient of variation of less than 10% for between-day as well as within-run imprecision. The lower limit of detection (LLOD) was 2.0 μg/mL and the lower limit of quantification (LLOQ) was 3.0 μg/mL. SCr concentrations were measured by kinetic Jaffe (compensated method) traceable to the IDMS reference method using AU5800 analyzer (Beckman coulter, Inc., Brea, CA, the USA). A coefficient variation was less than 3.0%, and the analytical measurement range (AMR) was 0.06 – 25.0 mg/dL. In case of BQL values, we treated them as observed values of extrapolation.

### Population pharmacokinetic modeling

For the analysis of vancomycin population pharmacokinetic parameters, nonlinear mixed-effect modeling was performed using NONMEM ver. 7.3 (Icon Development Solutions, Ellicott City, MD, USA) in conjunction with Perl Speaks NONMEM (PsN ver. 4.4.0), Pirana (ver. 2.9.7), and Xpose (ver. 4.0) contained in the R software package (ver. 3.5.0; http://www.r-project.org). The population analysis was conducted using the first-order conditional estimation method (FOCE) with ε–η interaction (FOCE INTER). One- and two-compartment models with first-order elimination were evaluated as potential structural pharmacokinetic models. The fixed-effect parameters were total clearance (CL) and volume of distribution (V) for the one-compartment model, and total CL, central V (V1), peripheral V (V2), and inter-compartmental CL (Q) for the two-compartment model. Possible correlations among the interindividual variability for pharmacokinetic parameters in the model were assessed by the OMEGA BLOCK functionality. Allometric scaling was applied to CL and isometric scaling was applied to V to standardize the pharmacokinetic parameters. The final allometric model was as follows:

3 $$\mathrm{CL}={\uptheta }_{CL}\times {(\frac{WTi}{70})}^{0.75}$$

4 $$\mathrm{V}={\uptheta }_{V}\times {(\frac{WTi}{70})}^{1}$$

where CL is the clearance in the ith individual with body weight WTi; θ_CL_ is the typical CL value in a standardized adult (70 kg); and θ_v_ is the typical V in a standardized adult (70 kg). Interindividual variability in the PK parameters was analyzed using exponential and power models. Covariance between interindividual variability was estimated using a variance–covariance matrix. Residual variability was attempted using additive, proportional, and combined models.

Covariates were investigated for the population modeling using linear, proportional, power, and exponential functions. Almost all patient characteristics were tried as covariates in the modeling process. When objective function value was decreased by at least 3.84 (χ^2^-test, P < 0.05) from the structural model during the forward selection, the covariate was included in the model. All covariates that met the criteria were added to develop the full model. Then, during backward elimination, any covariate with an increase of at least 6.64 (χ^2^-test, P < 0.01) was kept in the model. The categorical covariates tested were as follows: sex (where female = 1, male = 0), and types of delivery (where NsvD = 0, Csec = 1). The continuous covariates tested were body weight, blood urea nitrogen, SCr, protein, albumin, neutrophil, AST, ALT, CRP, WBC, height, PMA, PNA, CLcr, and gestational age.

### Model validation

Model validation was performed via three methods: graphical diagnostics using a goodness-of-fit plot, bootstrapping, and a pc-VPC. Individual observed vs. individual-predicted values and conditional weighted residual vs. time or population-predicted values or individual-predicted values were plotted using R for NONMEM. Bootstrap datasets were repeated 1,000 times using the final model; the pc-VPC was performed as a diagnostic plot.

### Simulation

Monte Carlo simulation was performed to predict vancomycin concentrations from the different dosing regimens to determine dose optimization for neonates. As PMA and CLcr were shown to be related to the population PK parameters of vancomycin in neonates, the simulation of vancomycin doses of 10 mg/kg q6h to q24h, 15 mg/kg q6h to q24h, and 20 mg/kg q6h to q24h were used to predict the vancomycin peak and trough concentrations according to PMA 28, 32, 36, 40, and 44 weeks and CLcr 30, 60, and 90 mL/min/1.73 m^2^. based on targeted efficacy reaching 400 ≤ AUC_0-24_ ≤ 600 when MIC assumed to be 1 mg/L as the update of therapeutic monitoring of vancomycin guideline of the American Society of Health-System Pharmacists^12,28,35–37^. Different from the conventional target of probability of target attainment (PTA) with 90% or higher when determining the PK/PD target of antibiotics^25^, the PTA of vancomycin based on the AUC/MIC ≥ 400 target was reported to be much lower which was only 49% of the standard dose of 1000 mg q12h in case of MRSA pathogens^38^. The target PTA of 400 ≤ AUC_0-24_ ≤ 600 was set to be higher than 30%. To reduce the confusion, the PTAs of AUC_0-24_ ≥ 400 was also calculated with the target ≥ 90%. If more than one regimen is recommended in the same situation, dosage of the lowest daily dose and more frequent dosing with lower peak concentrations among the same daily dose is chosen to reduce the toxicity in pediatrics (Suppl. Information).

## Supplementary Information

Supplementary Dataset 1.

Supplementary Dataset 2.

Supplementary Information.

Supplementary Dataset 3.

Supplementary Figures.
