# Supplementary material for: Population pharmacokinetics and dose optimization of vancomycin in neonates
Source: Sci Rep. 2021 Mar 17;11:6168. doi: 10.1038/s41598-021-85529-3 (PMC7969932; doi:10.1038/s41598-021-85529-3)
Supplement: Supplementary file 5 — Supplementary Figures. [file 41598_2021_85529_MOESM5_ESM.docx]

**Population pharmacokinetics and dose optimization of vancomycin in neonates**

Soon Min Lee^1^*, Seungwon Yang^2^*, Soyoung Kang^3^ and Min Jung Chang^2,3^

^1^Department of Pediatrics, Yonsei University College of Medicine, Seoul, Korea

^2^Department of Pharmacy and Yonsei Institute of Pharmaceutical Sciences, Yonsei University, Incheon, Republic of Korea

^3^Department of Pharmaceutical Medicine and Regulatory Science, Yonsei University, Incheon, Republic of Korea

* They did equal contribution to this work.


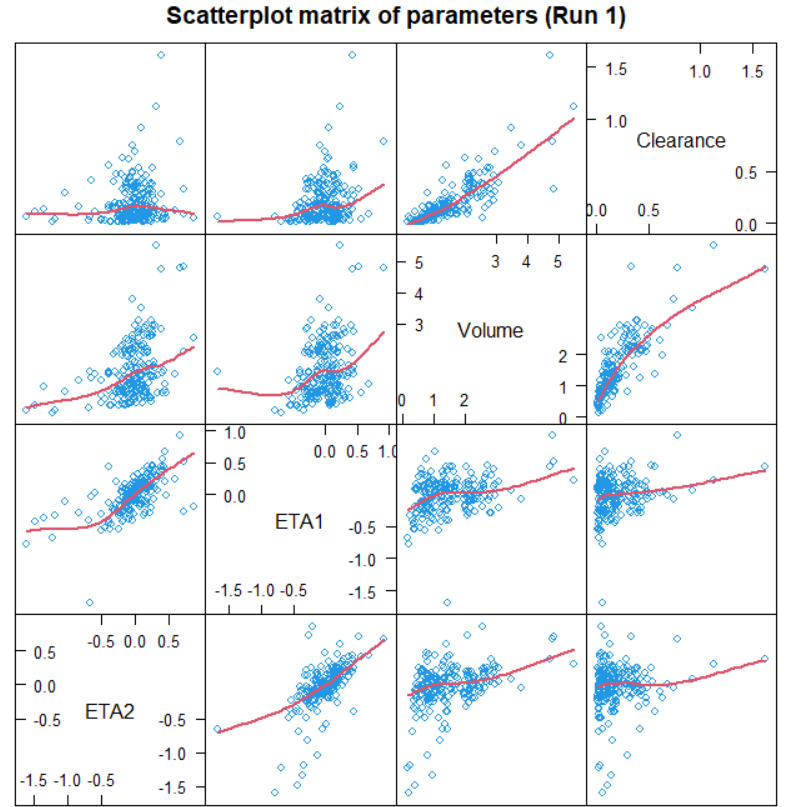


Figure S1. Scatterplot matrix of ETA


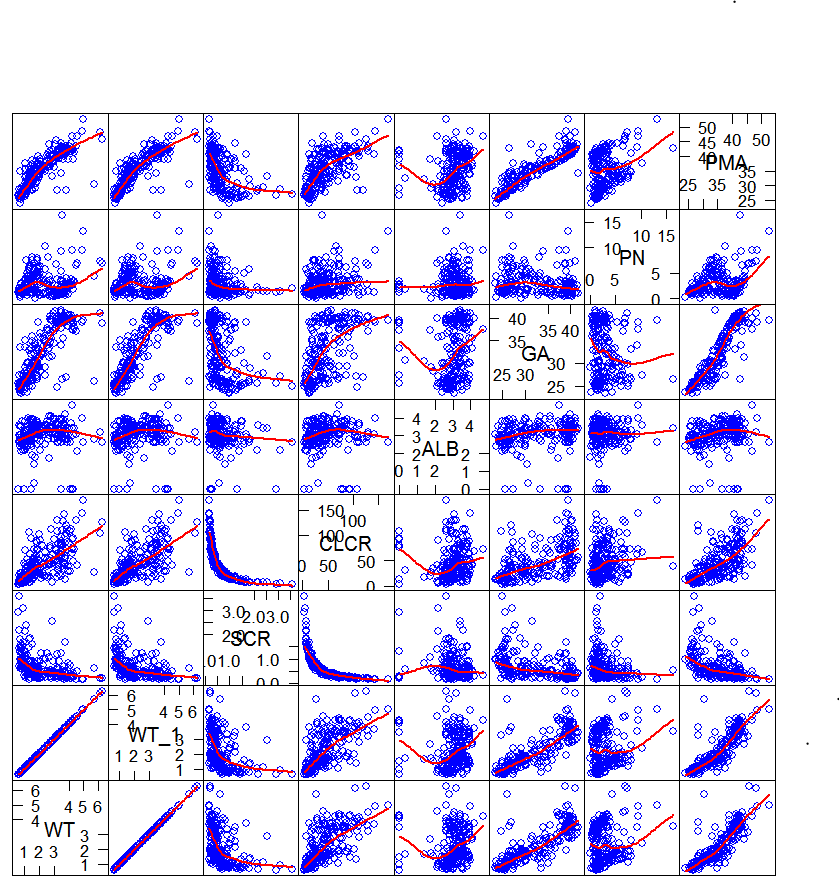


Figure S2. Scatterplot matrix of covariates

Figure 3. Scatterplot matrix of ETA
